# Supplementary material for: Nomogram Predicting In-Hospital Mortality in Patients with Myocardial Infarction Treated with Primary Coronary Interventions Based on Logistic and Angiographic Predictors
Source: Biomedicines. 2025 Mar 6;13(3):646. doi: 10.3390/biomedicines13030646 (PMC11940298; doi:10.3390/biomedicines13030646)
Supplement: Supplementary file 1 [file biomedicines-13-00646-s001.zip › biomedicines-3461429-supplementary.pdf]

Table S1. Missing values of variables.

Variables:	Population of patients treated with coronarography (N=603)	Population of patients treated with PCIs (N= 518)
General factors:		
Sex	0%	0%
Age	0%	0%
Spectrum of MI (STEMI/NSTEMI/UA)	0%	0%
Angiographic factors:		
Vascular access	0%	0%
Result of coronarography study	5.14%	0%
PCI on LM/proximal LAD	13.93%	0%
Restenosis in a DES in IRA	13.93%	0%
Post-PCI TIMI flow grade	14.1%	0%
Pre-dilatation with balloon (SC or NC)	14.76%	0.77%
Post-dilatation with NC balloon	14.60%	0.58%
PCI in bifurcation	13.93%	0%
Coronary artery calcifications in IRA	14.26%	0.19%
CA with subsequent CPR (CL stage)	0%	0%
Unsuccessful PCI	14.26%	0.19%
Logistical factors:		
Mode of presentation	0%	0%
Time of hospital admission	0%	0%
Time of PCI	0%	0%

Values are percentages (n). CA-cardiac arrest; CL-catheterisation laboratory; CPR-cardiopulmonary resuscitation; IRA-infarction-related artery; LAD-left anterior descending artery; LM-left main. MI-myocardial infarction; NC-non-compliant; PCI-primary coronary intervention; SC-semi-compliant; STEMI-ST-elevation myocardial infarction; TIMI -Thrombolysis in Myocardial Infarction flow grade.

Table S2. Comparison between derivation and validation sets

Variables	Derivation set (n=359)	Validation set (n=244)	P value
Mortality rate	5.57%	6.15%	0.766
General factors:			
Age (per 10-year increase)			
< 40 years old	1.39%	1.23%	0.686
40 – 49 years old	3.06%	3.69%	
50 – 59 years old	20.06%	18.85%	
60 – 69 years old	36.21%	37.30%	
70 – 79 years old	24.79%	20.49%	
89 – 89 years old	13.09%	17.62%	
≥ 90 years old	1.39%	0.82%	
Women	32.59%	34.84%	0.566
Men	67.41%	65.16%	
STEMI	34.54%	38.11%	0.369
NSTEMI/UA	65.46%	61.89%	

Angiographic factors:

Vascular access:			
Right radial artery	83.01%	85.66%	0.671
Left radial artery	16.16%	13.52%	
Right/left femoral artery	0.84%	0.82%	
Result of coronarography study:			
One-vessel disease	25.07%	25.33%	0.717
Double-vessel disease	26.82%	22.71%	
Multi-vessel disease with affected LM	35.57%	38.43%	
Multi-vessel disease without affected LM	12.54%	13.54%	
PCI on LM/proximal LAD	26.67%	21.57%	0.188
Restenosis in a DES in IRA	5.08%	2.94%	0.238
Post-PCI TIMI flow grades 0 -1	2.88%	1.94%	0.495
Post-PCI TIMI flow grades 2 – 3	97.12%	98.06%	
Pre-dilatation with balloon (SC or NC)	83.87%	85.29%	0.662
Post-dilatation with NC balloon	70.00%	74.15%	0.305
PCI in bifurcation	13.42%	12.14%	0.668
Coronary artery calcifications in IRA	15.34%	14.71%	0.844
CA with subsequent CPR (CL stage)	2.23%	1.64%	0.607
Unsuccessful PCI	2.56%	1.96%	0.657
Logistical factors:			
Mode of presentation			
Admission from home/public place	82.17%	85.25%	0.576
Admission from another hospital	13.37%	11.48%	
Admission from another department	4.46%	3.28%	
Time of hospital admission:			
Weekday from 8:00 to 14:00	37.88%	35.25%	0.583
Weekday from 14:00 to 22:00	25.63%	22.13%	
Weekday from 22:00 to 8:00	12.26%	15.98%	
Public holiday from 8:00 to 22:00	15.60%	17.62%	
Public holiday from 22:00 to 8:00	8.64%	9.02%	
Time of PCI:			
Weekday from 8:00 to 14:00	35.10%	37.30%	0.403
Weekday from 14:00 to 22:00	36.49%	9.51%	
Weekday from 22:00 to 8:00	7.80%	8.20%	
Public holiday from 8:00 to 22:00	15.88%	18.03%	
Public holiday from 22:00 to 8:00	4.74%	6.97%	

Values are percentages (n). CA-cardiac arrest; CL-catheterisation laboratory; CPR-cardiopulmonary resuscitation; IRA-infarction-related artery; LAD-left anterior descending artery; LM-left main. MI-myocardial infarction; NC-non-compliant; PCI-primary coronary intervention; SC-semi-compliant; STEMI-ST-elevation myocardial infarction; TIMI Thrombolysis in Myocardial Infarction flow grade.

Table S3. Clinical characteristics of the patients in the final derivation and validation sets according to their in-hospital mortality status after.

Variables	Derivation set (n=312)		Validation set (n=206)	
	Dead (n=19)	Alive (n=293)	Dead (n=12)	Alive (n=194)
**General factors:**				
Age (per 10-year increase)				
< 40 years old	20.00% (1)	80.00% (4)	0% (0)	100% (1)
40 – 49 years old	9.09% (1)	90.91% (10)	0% (0)	100% (8)
50 – 59 years old	0% (0)	100% (62)	10.26% (4)	89.74% (35)
60 – 69 years old	4.42% (5)	95.58% (108)	2.60% (2)	97.40% (75)
70 – 79 years old	6.11% (6)	91.89% (68)	7.50% (3)	92.50% (37)
89 – 89 years old	11.90% (5)	88.10% (37)	7.50% (3)	92.50% (37)
≥ 90 years old	20.00% (1)	80.00% (4)	100.00% (1)	0.00% (0)
Women	3.88% (4)	96.12% (99)	6.94% (5)	93.06% (67)
Men	7.18% (15)	92.82% (194)	5.22% (7)	94.78% (127)
STEMI	7.63% (9)	92.37% (109)	7.06% (6)	92.94% (79)
NSTEMI/UA	5.15% (10)	94.85% (184)	4.96% (6)	95.04% (115)
**Angiographic factors:**				
Vascular access:				
Right radial artery	4.67% (12)	95.33% (245)	4.00% (7)	96.00% (168)
Left radial artery	13.46% (7)	86.54% (45)	17.24% (5)	82.76% (24)
Right/left femoral artery	0% (0)	100% (3)	0% (0)	100% (2)
Result of coronarography study:				
One-vessel disease	2.47% (2)	97.53% (79)	7.55% (4)	92.45% (49)
Double-vessel disease	3.37% (3)	96.63% (86)	2.00% (1)	98.00% (49)
Multi-vessel disease with affected LM	7.55% (8)	92.45% (98)	3.66% (3)	96.34% (79)
Multi-vessel disease without affected LM	16.67% (6)	83.33% (30)	19.05% (4)	80.95% (17)
PCI on LM/proximal LAD	12.05%(10)	87.95% (73)	9.3% (4)	90.7% (39)
Restenosis in a DES in IRA	6.25% (1)	93.75% (15)	16.67% (1)	83.33% (5)
Post-PCI TIMI flow grades 0 -1	44.44% (4)	55.56% (5)	50.00% (2)	50.00% (2)
Post-PCI TIMI flow grades 2 – 3	4.95% (15)	95.05% (288)	4.95% (10)	95.05% (192)
Pre-dilatation with balloon (SC or NC)	6.18% (16)	93.82% (243)	5.75% (10)	94.25% (164)
Post-dilatation with NC balloon	3.70% (8)	96.30% (208)	3.95% (6)	96.05% (146)
PCI in bifurcation	2.38% (1)	97.62% (41)	4.00% (1)	96.00% (24)
Coronary artery calcifications in IRA	12.50% (6)	87.50% (42)	6.67% (2)	93.33% (28)
CA with subsequent CPR (CL stage)	42.86% (3)	57.14% (4)	50.00% (2)	50.00% (2)
Unsuccessful PCI	50.00% (4)	50.00% (4)	50.00% (2)	50.00% (2)
**Logistical factors:**				
Mode of presentation				
Admission from home/public place	4.65% (12)	95.35% (246)	6.21% (11)	93.79% (166)
Admission from another hospital	9.52% (4)	90.48% (38)	0.00% (0)	100% (23)
Admission from another department	25.00% (3)	75.00% (9)	16.67% (1)	83.33% (5)
Time of hospital admission:				
Weekday from 8:00 to 14:00	2.61% (3)	97.39% (112)	2.82% (2)	97.18% (69)
Weekday from 14:00 to 22:00	12.20% (10)	87.80% (72)	4.76% (2)	95.24% (40)
Weekday from 22:00 to 8:00	8.11% (3)	91.89% (34)	8.11% (3)	91.89% (34)

Public holiday from 8:00 to 22:00	4.00% (2)	96.00% (48)	2.78% (1)	97.22% (35)
Public holiday from 22:00 to 8:00	3.57% (1)	96.43% (27)	20.00% (4)	80.00% (16)
Time of PCI:				
Weekday from 8:00 to 14:00	1.94% (2)	98.06% (101)	5.26% (4)	94.74% (72)
Weekday from 14:00 to 22:00	8.85% (10)	91.15% (103)	5.00% (3)	95.00% (57)
Weekday from 22:00 to 8:00	15.38% (4)	84.62% (22)	10.53% (2)	89.47% (17)
Public holiday from 8:00 to 22:00	5.56% (3)	94.44% (51)	5.71% (2)	94.29% (33)
Public holiday from 22:00 to 8:00	0% (0)	100% (16)	6.25% (1)	93.75% (15)

Values are percentages (n). CA-cardiac arrest; CL-catheterisation laboratory; CPR-cardiopulmonary resuscitation; IRA-infarction-related artery; LAD-left anterior descending artery; LM-left main, MI-myocardial infarction; NC-non-compliant; PCI-primary coronary intervention; SC-semi-compliant; STEMI-ST-elevation myocardial infarction; TIMI-Thrombolysis in Myocardial Infarction flow grade.
